# Pulmonary Shunt in Critical Care: A Comprehensive Review of Pathophysiology, Diagnosis, and Management Strategies

**DOI:** 10.7759/cureus.68505

**Published:** 2024-09-03

**Authors:** Sharayu Paunikar, Vivek Chakole

**Affiliations:** 1 Anesthesiology, Jawaharlal Nehru Medical College, Datta Meghe Institute of Higher Education and Research, Wardha, IND

**Keywords:** ventilation-perfusion mismatch, management strategies, diagnostic techniques, pathophysiology, critical care, pulmonary shunt

## Abstract

Pulmonary shunt, an abnormal passage of blood through the pulmonary circulation without adequate gas exchange, poses significant challenges in critical care. This comprehensive review explores the pathophysiology, diagnostic approaches, and management strategies associated with pulmonary shunt. Pulmonary shunts are classified into anatomical and physiological types, each with distinct mechanisms and implications for gas exchange. Anatomical shunts result from structural heart defects, while physiological shunts arise from ventilation-perfusion (V/Q) mismatches. Both conditions can significantly impair oxygenation and contribute to multi-organ dysfunction. This review delves into various diagnostic modalities, including clinical assessment, imaging techniques such as chest X-ray and CT scans, and advanced diagnostic methods such as V/Q scanning and echocardiography. Challenges in diagnosing pulmonary shunt are discussed, emphasizing the limitations of current tools and the need for accurate differentiation of shunt types. Management strategies are examined, covering pharmacological interventions, non-pharmacological treatments such as mechanical ventilation and prone positioning, and surgical options. Emerging therapies and innovations in treatment are also highlighted. Special considerations are given to different patient populations, including pediatric and elderly patients and those with multiple comorbidities. This review concludes with an analysis of the prognosis and outcomes associated with pulmonary shunt, focusing on short-term and long-term impacts on survival and quality of life. This review aims to enhance understanding and guide effective management practices for pulmonary shunt in critical care settings by synthesizing current knowledge and identifying areas for further research.

## Introduction and background

Pulmonary shunt is characterized by the abnormal passage of blood through the pulmonary circulation without undergoing adequate gas exchange in the lungs. This phenomenon can be divided into anatomical and physiological types [1]. Anatomical shunts arise from structural heart defects, such as a ventricular septal defect or patent ductus arteriosus, which cause blood to flow directly from the right to the left side of the heart or vice versa. Physiological shunts, however, result from a mismatch between ventilation and perfusion, where certain lung areas receive blood flow but are not effectively ventilated, leading to impaired oxygenation. Both shunts compromise the efficiency of oxygen uptake and carbon dioxide removal, significantly affecting systemic oxygen delivery and overall patient outcomes [2].

In critical care settings, the significance of pulmonary shunt is considerable due to its impact on respiratory and circulatory function. In critically ill patients, the presence of a pulmonary shunt can exacerbate hypoxemia and contribute to multi-organ dysfunction, making timely diagnosis and effective management essential. Conditions such as acute respiratory distress syndrome (ARDS), severe pneumonia, and heart failure can be complicated by shunting, underscoring the importance of addressing this issue to improve patient outcomes, reduce mortality, and enhance the overall quality of care [1].

This review aims to thoroughly examine pulmonary shunt in critical care, focusing on its pathophysiology, diagnostic approaches, and management strategies. By consolidating current knowledge and highlighting advancements in the field, this review seeks to offer a comprehensive resource for clinicians, researchers, and healthcare professionals involved in the care of critically ill patients. This review will explore several key areas: the underlying mechanisms of pulmonary shunting (including anatomical and physiological aspects), diagnostic techniques and tools used to identify shunts, current management approaches (including pharmacological, non-pharmacological, and surgical options), and emerging therapies. Additionally, this review will address special considerations for different patient populations, such as pediatric and elderly patients and those with multiple comorbidities. It will also analyze the short-term and long-term outcomes associated with pulmonary shunt, including its impact on patient survival and quality of life. This review aims to enhance understanding and guide effective management practices for pulmonary shunt in critical care by covering these aspects.

## Review

Pathophysiology of pulmonary shunt

Pulmonary shunting can be categorized into two primary types: anatomical and physiological. Anatomical shunts involve structural abnormalities that allow deoxygenated blood to mix with oxygenated blood without passing through the alveoli. A typical example is a right-to-left shunt, which can occur in congenital heart defects such as patent ductus arteriosus, ventricular septal, and atrial septal defects. In these conditions, blood flows from the right side of the heart to the left side, bypassing the lungs entirely and causing systemic hypoxemia [1]. In contrast, physiological shunts occur when blood bypasses ventilated alveoli, resulting in a ventilation-perfusion (V/Q) mismatch. This shunt is often seen in conditions such as pneumonia and acute respiratory distress syndrome (ARDS), where particular alveoli are perfused but not adequately ventilated. In these situations, the affected lung areas cannot participate in gas exchange, leading to reduced oxygen levels in the arterial blood [3]. Several factors can contribute to developing pulmonary shunts, including cardiac abnormalities, respiratory conditions, and vascular changes. Cardiac abnormalities, especially congenital heart defects, can create pathways for deoxygenated blood to enter the systemic circulation without passing through the lungs. Additionally, right heart failure can increase pulmonary pressures, further exacerbating shunting [4].

Respiratory conditions also significantly contribute to the development of pulmonary shunts. For example, ARDS and pneumonia can cause alveolar flooding or collapse, which prevents effective gas exchange and results in physiological shunting. Furthermore, vascular changes, such as pulmonary embolism, can obstruct blood flow, creating areas of the lung that are perfused but not ventilated, thus contributing to shunting. Similarly, pulmonary hypertension increases vascular resistance, further complicating the dynamics of pulmonary blood flow [4]. The presence of pulmonary shunts profoundly impacts gas exchange and oxygenation. When deoxygenated blood bypasses ventilated alveoli, it results in hypoxemia, a condition characterized by low oxygen levels in the blood. The severity of hypoxemia is directly related to the degree of shunting; as more blood bypasses the lungs, the oxygen content in the arterial blood decreases. It can have significant clinical implications, particularly for critically ill patients [3].

**Figure 1:**
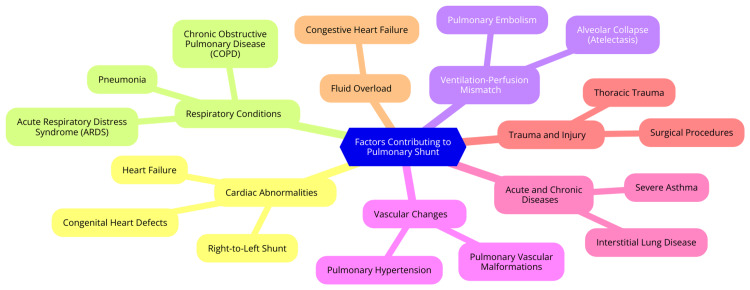
Factors contributing to pulmonary shunt Image Credit: Dr. Sharayu Paunikar

Moreover, pulmonary shunting can worsen other critical conditions, such as respiratory failure, by impairing overall oxygenation. Patients with pre-existing respiratory issues may experience poorer outcomes due to the additional burden of shunting. Understanding pulmonary shunts' mechanisms and contributing factors is essential for healthcare providers in critical care settings, as it enables them to implement effective management strategies to mitigate the impact on gas exchange and improve patient outcomes [5]. Figure 1 illustrates the factors contributing to pulmonary shunt.

Diagnosis of pulmonary shunt

Diagnosing a pulmonary shunt involves a comprehensive approach that combines clinical assessment, diagnostic imaging, and advanced diagnostic techniques. Each of these elements plays a critical role in identifying the presence and extent of shunting, which is essential for effective management [5]. The clinical assessment begins with thoroughly evaluating the patient's symptoms and overall clinical presentation. Patients with a pulmonary shunt often exhibit signs of respiratory distress, such as shortness of breath, cyanosis, and exercise intolerance. The severity of these symptoms can vary depending on the degree of shunting and the underlying cause. A detailed physical examination may reveal additional findings, including clubbing of the fingers and toes, spider angiomas, and signs of right heart failure, which could indicate chronic hypoxemia or elevated pulmonary vascular pressures [6]. Diagnostic imaging is a crucial part of the diagnostic process. A chest X-ray is typically the initial imaging modality used and can detect underlying lung pathologies that contribute to shunting, such as atelectasis, consolidation, or pulmonary edema [7]. For a more comprehensive evaluation, a computed tomography (CT) scan provides high-resolution images of the lung parenchyma and vasculature, aiding in identifying structural abnormalities that could lead to shunting. In some instances, magnetic resonance imaging (MRI) may assess complex congenital heart diseases and pulmonary vascular malformations, providing additional insights that other imaging modalities may not offer [8].

Beyond standard imaging, advanced diagnostic techniques are essential for a more precise diagnosis of a pulmonary shunt. V/Q scanning is a specialized test that assesses air and blood flow distribution within the lungs, helping to identify perfused but not ventilated areas, indicating shunting [9]. Echocardiography, including transthoracic and transesophageal approaches, is especially useful for detecting intrapulmonary right-to-left shunts, such as those resulting from pulmonary arteriovenous malformations. Additionally, arterial blood gas (ABG) analysis is critical for evaluating the patient's oxygenation status. It allows for calculating the shunt fraction, providing quantitative data to inform treatment decisions [9]. Despite the availability of various diagnostic tools, diagnosing a pulmonary shunt presents significant challenges and limitations. Each technique has its strengths and weaknesses concerning sensitivity and specificity [10]. For example, while chest X-rays can indicate the presence of lung pathology, they may not definitively confirm the presence of shunting.

Moreover, differentiating between physiological and pathological shunts can be particularly difficult, as the clinical presentation may overlap, and imaging findings might not always clearly distinguish the type of shunt. These complexities highlight the need for a comprehensive and nuanced diagnostic approach that integrates clinical findings with advanced diagnostic techniques to achieve the most accurate assessment possible [11]. Table 1 summarizes the diagnostic methods for pulmonary shunt [12-16].

**Table 1:** Diagnostic methods for pulmonary shunt CT: Computed tomography; MRI: Magnetic resonance imaging; V/Q: Ventilation-perfusion; ABG: Arterial blood gas

Diagnostic Method	Description	Strengths	Limitations
Clinical Assessment	Initial evaluation based on symptoms, physical examination, and patient history	Provides quick, preliminary information	Lack of specificity and be dependent on clinical judgment
Chest X-ray	Imaging technique to visualize lung structure and potential abnormalities	Widely available; helpful in identifying gross abnormalities	Limited in detecting subtle shunts or assessing functional status
CT Scan	Detailed cross-sectional imaging for a more precise view of lung and heart structures	High-resolution imaging; better detection of structural issues	Radiation exposure not always differentiating between shunt types
MRI	Non-invasive imaging to provide detailed visualization of soft tissues	No radiation; detailed images of soft tissue and vascular structures	Higher cost; less available than CT; limited in assessing lung function
V/Q Scanning	Imaging test to assess the distribution of ventilation and perfusion in the lungs	Effective for detecting physiological shunts; helps identify ventilation-perfusion mismatches	Limited in detecting anatomical shunts; may require comparison with other tests
Echocardiography	Ultrasound imaging of the heart to evaluate structural abnormalities and blood flow	Non-invasive; useful for detecting anatomical shunts and assessing cardiac function	May not visualize all shunt types; operator-dependent
ABG Analysis	Blood tests to measure gas levels in blood and calculate shunt fractions	Provides quantitative data on gas exchange, which helps assess the impact of shunts	Does not directly visualize shunts and requires interpretation in the context of clinical findings

Management strategies for pulmonary shunt

The primary goals in managing a pulmonary shunt include improving oxygenation, preventing complications associated with hypoxemia, and addressing the underlying causes of the shunt. It requires a thorough assessment of the patient's hemodynamics and pulmonary function to tailor interventions appropriately. Continuous monitoring of vital signs, arterial blood gases, and oxygen saturation is crucial for effective management. Supportive care may involve fluid management, nutritional support, and advanced monitoring techniques to evaluate pulmonary function and the severity of the shunt [17]. Pharmacological treatments often address the underlying causes of pulmonary shunting, such as pulmonary hypertension. Medications may include pulmonary vasodilators (which help reduce pulmonary vascular resistance (PVR) and improve blood flow through the lungs), diuretics (which manage fluid overload, particularly in cases of pulmonary edema), and inotropes (which can enhance cardiac output in patients with heart failure that contribute to shunting). Specific vasodilators, such as phosphodiesterase-5 inhibitors and endothelin receptor antagonists, may manage pulmonary arterial hypertension (PAH) associated with shunts. Diuretics assist in reducing pulmonary congestion, while inotropes may be necessary when cardiac output is compromised [18].

Mechanical ventilation settings may be adjusted to optimize oxygenation and reduce the effects of shunting. Strategies include low tidal volume ventilation, which can help minimize ventilator-induced lung injury and high positive end-expiratory pressure (PEEP), which can improve the recruitment of collapsed alveoli and enhance gas exchange. Prone positioning has been demonstrated to improve oxygenation in patients with severe respiratory failure, particularly those with ARDS, as it can help redistribute blood flow and improve V/Q matching. Supplemental oxygen is essential in managing hypoxemia associated with pulmonary shunts, and high-flow nasal cannula or non-invasive ventilation may be employed to enhance oxygen delivery [19]. Surgical intervention may be indicated in cases where anatomical shunts are present, such as congenital heart defects. Surgery timing is critical and often determined by the degree of PVR and the patient's overall clinical condition. Procedures such as atrial septostomy may be used in severe cases of pulmonary hypertension to create a controlled shunt, alleviating right ventricular strain and improving cardiac output. This approach is particularly relevant in patients with Eisenmenger syndrome [19].

Research into new pharmacological agents and therapies is ongoing, focusing on targeted treatments for pulmonary hypertension. These may include novel vasodilators and anti-remodeling agents that aim to reverse pathological pulmonary vascular changes. Technological advancements, such as improved imaging modalities and minimally invasive surgical techniques, enhance the ability to diagnose and treat pulmonary shunts more effectively. Innovations in monitoring systems, including non-invasive methods for assessing shunt severity, are also being explored to facilitate better clinical decision-making [20]. Table 2 summarizes the management strategies for pulmonary shunts [20-25].

**Table 2:** Management strategies for pulmonary shunt ARDS: Acute respiratory distress syndrome

Management Strategy	Description	Indications	Advantages	Limitations
Pharmacological Interventions	Medications to manage underlying conditions contributing to shunt	Common in conditions, such as heart failure or pulmonary hypertension	Address underlying causes and symptoms and is less invasive	Have side effects and limited efficacy in severe cases
Vasodilators	Medications to dilate blood vessels improve blood flow and reduce pressure	Used for conditions, such as pulmonary hypertension	Improve hemodynamics and reduce right heart strain	Risk of hypotension and potential interaction with other medications
Diuretics	Medications to reduce fluid overload decrease the burden on the heart and lungs	Helpful in cases with fluid retention, such as in heart failure	Reduce fluid buildup, improving pulmonary and cardiac function	Potential for electrolyte imbalances and dehydration
Inotropes	Drugs that increase the force of heart muscle contraction	Indicated in cases of low cardiac output or severe heart failure	Improve cardiac output and tissue perfusion	Risk of arrhythmias and potential worsening of heart failure in some cases
Mechanical Ventilation	Use of ventilators to assist or control breathing	Applied in cases of severe hypoxemia or respiratory distress	Improves oxygenation and ventilation and customizable to patient needs	Risk of ventilator-associated complications; may not address underlying shunt directly
Prone Positioning	Positioning the patient face down to improve ventilation and perfusion matching	Beneficial in ARDS and severe hypoxemia cases	Enhances ventilation and reduces shunting effects	Unsuitable for all patients; requires careful monitoring and management
Oxygen Therapy	Administration of supplemental oxygen to increase oxygen levels in the blood	Used to address hypoxemia resulting from shunt	Improves oxygenation and relieves hypoxia.	Does not address the underlying cause of shunting; the potential for oxygen toxicity in extreme cases
Surgical Correction	Surgical procedures to correct anatomical shunts or structural defects	Indicated for congenital heart defects or severe anatomical shunts	Provide a definitive solution for anatomical shunts	Involves surgical risks and may require long recovery periods
Interventional Cardiology	Techniques such as atrial septostomy or device closure to manage shunts	Used in cases where anatomical correction is needed, such as atrial septal defects	Minimally invasive compared to open surgery; effective for specific defects	Not suitable for all patients because of the risks of procedure-related complications
Emerging Therapies	New and experimental treatments aimed at improving shunt management	Applied in advanced or refractory cases	Potential for innovative solutions and improved outcomes	Limited availability and unproven long-term efficacy

Special considerations

Pediatric Population

Diagnosing and managing pulmonary shunts in the pediatric population presents unique challenges. Children, notably neonates and infants, have different lung mechanics and anatomical structures compared to adults, which complicates the assessment of shunting. Additionally, congenital heart defects, which are more common in this age group, can result in significant anatomical shunts that require careful evaluation and management. Young patients often cannot clearly articulate their symptoms, making it difficult for clinicians to assess the presence and severity of respiratory distress or hypoxemia. As a result, healthcare providers must rely heavily on physical examinations, clinical observations, and continuous monitoring to detect signs of shunting and hypoxemia [25].

Furthermore, pediatric patients have different baseline values for arterial blood gases and oxygen saturation, necessitating age-specific reference ranges for accurate diagnosis. Management strategies must also be tailored for this population, as children may respond differently to medications and interventions than adults. Dosing and treatment protocols must be meticulously calculated based on the child’s weight and developmental stage to ensure safety and efficacy. Non-invasive ventilation strategies are often preferred in pediatric patients to minimize the risk of barotrauma or volutrauma due to their more delicate lung structures and higher susceptibility to injury [26].

Elderly Population

The elderly population presents a distinct set of challenges in managing pulmonary shunts. Age-related physiological changes, such as reduced lung compliance, decreased respiratory muscle strength, and a diminished alveolar surface area, can worsen the effects of pulmonary shunting. Additionally, older adults often have a reduced physiological reserve, making them more vulnerable to hypoxemia and respiratory failure. The management of pulmonary shunts in this demographic is further complicated by the presence of multiple comorbidities, including chronic obstructive pulmonary disease (COPD), heart failure, and pulmonary hypertension, all of which can affect both the presentation and progression of the shunt [26]. These comorbid conditions may alter the response to treatments, requiring a multidisciplinary approach to care. Medication dosing must be carefully adjusted, as elderly patients often exhibit altered pharmacokinetics and pharmacodynamics, increasing their sensitivity to certain drugs. Therefore, a more conservative management approach is frequently warranted, focusing on optimizing oxygenation while minimizing the risks associated with invasive procedures. Additionally, palliative care considerations should be incorporated into the treatment plan, especially for patients with advanced age and a poor prognosis, to ensure that care is aligned with the patient's overall goals and quality of life [27].

Impact of Comorbidities

Patients with multiple underlying conditions present a complex clinical profile that complicates both the diagnosis and management of pulmonary shunts. Comorbidities such as diabetes, obesity, and cardiovascular diseases can exacerbate respiratory issues, increasing the complexity of managing pulmonary shunting. An interdisciplinary approach is essential for effective management, necessitating collaboration among various specialties, including pulmonology, cardiology, and critical care, to comprehensively address the multifaceted needs of these patients [28]. Developing tailored treatment plans is crucial, as these plans must consider the severity of each comorbidity and its specific impact on pulmonary function. It often involves optimizing underlying conditions, such as better control of heart failure or meticulous diabetes management, to reduce their adverse effects on respiratory status. Continuous monitoring is vital to evaluate interventions' effectiveness and make necessary real-time adjustments to the treatment plan. Regular follow-up is also essential for the early identification of potential complications, thereby improving overall outcomes for patients with pulmonary shunts [28].

Prognosis and outcomes

Short-Term Outcomes

A pulmonary shunt in critical care settings can significantly influence immediate patient management. Patients with pulmonary shunts often require intensive monitoring and intervention due to an increased risk of hypoxemia and other complications. For example, in the immediate postoperative period following procedures such as the Blalock-Taussig shunt, meticulous management of hemodynamics is essential. Monitoring parameters such as blood pressure and lactate levels is crucial to evaluate the effectiveness of the intervention and the patient's response to treatment. Due to the critical nature of these cases, a multidisciplinary approach is necessary to ensure optimal outcomes [29]. Recovery following procedures involving pulmonary shunts can vary significantly among patients.

In a cohort of neonates who underwent the modified Blalock-Taussig shunt, the average duration of mechanical ventilation was approximately 3.9 days, with a mortality rate of 13.6% within the first month post-surgery, primarily due to shunt-related complications such as thrombosis. These complications, including shunt failure, may require additional surgical intervention. It underscores the importance of vigilant postoperative care and follow-up, as the risk of complications can substantially impact short-term recovery and overall patient outcomes [30].

Long-Term Outcomes

Long-term outcomes for patients with pulmonary shunts are shaped by various factors, including the underlying condition prompting the shunt and the timing and type of surgical interventions. In patients with congenital heart diseases who have undergone shunt repairs, there are often notable improvements in functional status and quality of life postoperatively. For instance, a study found that patients who received targeted pulmonary arterial therapy before shunt closure showed significant enhancements in World Health Organization functional class scores, reflecting better overall health and improved quality of life. It highlights the critical role of individualized treatment plans in optimizing long-term outcomes [31,32]. The prognosis for patients with pulmonary shunts is also heavily dependent on the effective management of PVR and the prevention of complications such as PAH. Studies indicate that early intervention and diligent monitoring can lead to positive outcomes, with many patients experiencing significant gains in functional status and extended survival rates. However, persistent PAH following shunt closure is associated with a poorer prognosis, underscoring the necessity of timely and targeted management strategies to enhance long-term outcomes and reduce the risk of adverse events [33,34].

## Conclusions

In conclusion, pulmonary shunt represents a complex and critical challenge in managing critically ill patients. Understanding the intricate mechanisms behind both anatomical and physiological shunting is essential for accurate diagnosis and effective treatment. This review has highlighted the importance of timely and precise diagnostic techniques, such as advanced imaging and diagnostic tools, which are crucial for identifying the presence and extent of shunting. Management strategies, ranging from pharmacological interventions to advanced surgical procedures, are pivotal in addressing the underlying causes and mitigating the impact of shunt-related complications. Special considerations for diverse patient populations further underscore the need for individualized approaches to care. Ultimately, a comprehensive understanding of pulmonary shunt and its management can significantly improve patient outcomes, enhance the quality of care in critical settings, and guide future research and innovations in the field. By integrating current knowledge and addressing gaps, this review aims to support clinicians in optimizing care for patients with pulmonary shunt and contribute to advancements in critical care practices.
